# Inhibition of inflammatory liver injury by the HMGB1-A box through HMGB1/TLR-4/NF-κB signaling in an acute liver failure mouse model

**DOI:** 10.3389/fphar.2022.990087

**Published:** 2022-10-14

**Authors:** Lidan Luo, Shuai Wang, Bohao Chen, Mei Zhong, Ruili Du, ChunShan Wei, Furong Huang, Xinhui Kou, Yufeng Xing, Guangdong Tong

**Affiliations:** ^1^ Department of Hepatology, The Fourth Clinical Medical College of Guangzhou University of Traditional Chinese Medicine, Shenzhen, China; ^2^ Department of Hepatology, Shenzhen Traditional Chinese Medicine Hospital Affiliated to Nanjing University of Chinese Medicine, Shenzhen, China; ^3^ Faculty of Chinese Medicine and State Key Laboratory of Quality Research in Chinese Medicine, Macau University of Science and Technology, Macao, China

**Keywords:** acute liver failure, HMGB1-A box, TLR-4/NF-κB signaling, extracellular HMGB1, inflammatory injury

## Abstract

We aimed to investigate the preventive effect of high mobility group box 1 (HMGB1)-A box and the mechanism by which it alleviates inflammatory injury in acute liver failure (ALF) by inhibiting the extracellular release of HMGB1. BALB/c mice were intraperitoneally (i.p.) administered LPS/D-GalN to establish an ALF mouse model. HMGB1-A box was administered (i.p.) 1 h before establishing the ALF mouse model. The levels of extracellularly released HMGB1, TLR-4/NF-κB signaling molecules, the proinflammatory cytokines TNF-α, IL-1β, and IL-6 and COX-2 were measured in the liver tissue and/or serum by Immunohistochemistry, Western blotting and Enzyme-linked immunosorbent assay (ELISA). The levels of extracellularly released HMGB1, TLR-4/NF-κB signaling molecules and proinflammatory cytokines were measured in Huh7 cells as well as LPS- and/or HMGB1-A box treatment by confocal microscopy, Western blotting and ELISA. In the ALF mouse model, the levels of HMGB1 were significantly increased both in the liver and serum, TLR-4/NF-κB signaling molecules and proinflammatory cytokines also was upregulated. Notably, HMGB1-A box could reverse these changes. HMGB1-A box could also cause these changes in LPS-induced Huh7 cells. HMGB1-A box played a protective role by inhibiting inflammatory liver injury *via* the regulation of HMGB1/TLR-4/NF-κB signaling in the LPS/D-GaIN-induced ALF mouse model, which may be related to inhibiting the extracellular release of HMGB1.

## Introduction

Acute liver failure (ALF) refers to massive necrosis or severe damage of hepatocytes and is caused by various conditions, resulting in clinical syndromes such as jaundice, ascites, hepatic encephalopathy and coagulation dysfunction, which are typically the primary manifestations (Grek and Arasi, 2016). Acute liver injury (ALI) is an early manifestation and an important feature in ALF. Owing to the massive necrosis of hepatocytes and the rapidly accelerated inflammatory response induced by ALI, the mortality rate of ALF is as high as 80%–90% (Fyfe et al., 2018; Zhang et al., 2020). Due to the severity and heterogeneity of ALF, there are very limited treatment options (Stravitz and Lee, 2019).

High mobility group box 1 (HMGB1) is a danger signal molecule. When the body is exposed to exogenous stimuli or endogenous damage, HMGB1 acts as a stress signal and an inflammatory mediator and is actively or passively released extracellularly by macrophages/monocytes or necrotic cells (Andersson et al., 2002). Extracellular HMGB1 can accelerate foam formation and macrophage apoptosis *via* the ERS/CHOP signaling pathway in OxLDL-induced RAW264.7 cells (Wu et al., 2018). Extracellular HMGB1 can also aggravate the inflammatory damage of cardiomyocytes by regulating the JNK/BAX signaling pathway in the rat myocardial ischemia–reperfusion model (Zhai et al., 2012). Furthermore, HMGB1 can upregulate TNF-α, IL-1β, and IL-18 to induce an inflammatory response in the cells of multiple organs, such as brain (Nishibori et al., 2020), lungs (Ding et al., 2017) and liver (Wang et al., 2020).

Box A and box B are two domains in the functional structure of HMGB1. Evidence indicates that the region of HMGB1 involved in inflammatory processes/signaling is primarily located in box B domain. box A domain is similar to box B domain, but it has no function in inducing inflammation. HMGB1-A box (Box A) has been reported to inhibit the inflammatory activity of HMGB1 by binding competitively with it. Intrathecal injection of recombinant HMGB1-A box protein could effectively prevent the activity of extracellular HMGB1 and reverse mechanical hypersensitivity in a collagen antibody-induced arthritis mouse model (Agalave et al., 2014). Moreover, intranasal infusion of recombinant HMGB1-A box could significantly reduce the infarct volume of stroke and a series of inflammatory reactions induced by LPS in a post-stroke infection rat model (Kim et al., 2018). In addition, HMGB1-A box could inhibit proinflammatory cytokines, such as TNF-α, by LPS induction *via* binding to heparin complex in an ALI mouse model (Kim et al., 2015).

The molecular mechanism by which HMGB1-A box inhibits inflammatory injury in ALF has rarely been investigated. Hence, to provide a scientific and theoretical basis for ALF treatment, this study investigated how HMGB1-A box alleviates and reduces ALI-induced liver inflammation by inhibiting the extracellular release of HMGB1.

## Materials and methods

### Animals and drugs

6–8 weeks BALB/c male mice (weighing 18–25 g) were purchased from Guangdong Medical Laboratory Animal Center (Guangzhou, China). All animal experiments were approved by the Animal Care and Use Committee of the Fourth Clinical Medical College of Guangzhou University of Traditional Chinese Medicine. The animals were raised according to the National Health Guidelines for the Care and Use of Laboratory Animals.

### LPS/D-GalN-induced ALF mouse model and high mobility group box 1-A box administration

The LPS/D-GalN-induced ALF mouse model has been described previously (Lei et al., 2015). Briefly, BALB/c mice were intraperitoneally (i.p.) administered LPS (from *Escherichia coli* 0111:B4) (10 μg/kg; L4391, Sigma-Aldrich) and D-GalN (600 mg/kg; G0500, Sigma-Aldrich) and were sacrificed 3, 12, and 24 h after administration. HMGB1-A box (400 μg; HM-014, HMGBiotech) was i.p. Administered 1 h before establishing the ALF mouse model and mice were sacrificed 24 h after establishing the ALF mouse model. Serum and liver tissues of all mice were collected and stored at −80°C until analysis.

### Hematoxylin and eosin and masson staining

Liver tissues were fixed in 4% paraformaldehyde (PFA; BL539A, BioSharp), embedded in paraffin and cut into 4-μm thick sections using a microtome. The sections were incubated overnight at 37°C and then for 2 h at 75°C. The deparaffinized sections were used for hematoxylin and eosin (H&E) and Masson staining and observed under a microscope (Axio Imager M2, Carl Zeiss).

### Immunohistochemistry

Liver sections were deparaffinized, rehydrated and incubated with anti-HMGB1 antibody (1:200, ab18256, Abcam) overnight at 4°C. Subsequently, they were washed and incubated with anti-rabbit IgG (1:1000, ab6721, Abcam) for 30 min at room temperature. The sections were stained with diaminobenzidine (DAB-0031, Maixin Biotechnology), counterstained with hematoxylin and examined under a microscope (Axio Imager M2, Carl Zeiss).

### Measurement of serum aminotransferase activity

Blood samples were centrifuged at 845 × g for 15 min at 4°C to separate the serum. Serum biochemical indices of alanine aminotransferase (ALT), aspartate aminotransferase (AST) and total bilirubin (TBIL) levels were measured using a multi-parameter analyzer (AU 5400, Olympus, Japan).

### Cell culture

Huh7 human hepatoma cell line was purchased from Procell Life Science and Technology (Wuhan, China). The cells were cultured in Dulbecco’s modified Eagle medium (DMEM; C11995500BT, Gibco) supplemented with 10% fetal bovine serum (10099141C, Gibco) and 1% penicillin–streptomycin (15140122, Gibco) in a humidified incubator with 5% CO_2_ at 37°C.

### Treatment with LPS and high mobility group box 1-A box

Huh7 cells were treated with 1000 ng/ml of LPS for 3, 9, 16, 24, and 48 h in serum-free DMEM or cotreated with HMGB1-A box (50 or 100 ng/ml) for 24 h. The cells were prepared at the indicated time points for experiments.

### Extraction of nuclear, cytoplasmic and extracellular proteins

Nuclear and cytoplasmic proteins were extracted using nuclear and cytoplasmic extraction reagents (78,835, Thermo Fisher Scientific, United States) according to the manufacturer’s instructions. The protocol was modified depending on the cell pellet volume. The volume ratio of CER I:CER II:NER reagents was maintained at 200:11:100 μl, respectively. Proteins in the LPS- and/or HMGB1-A box–treated media, i.e., extracellular proteins, were extracted after 24 h and concentrated from 500 to 20 µl using Centricon 10 (Centricon YM-10, Millipore).

### Cell viability assay

The viability of Huh7 cells was assessed using 3-[4,5-dimethylthiazol-3-yl] 2,5-diphenyltetrazolium bromide (MTT) after LPS treatment. Briefly, Huh7 cells (4 × 10^4^) were seeded into 24-well plates and cultured overnight. After treatment with LPS and/or HMGB1-A box, the cells were incubated with thiazolyl blue tetrazolium bromide (500 μg/ml; M2128, Sigma) for 1 h and the produced formazan was solubilized using DMSO (200 μl; D4540, Sigma). Subsequently, absorbance was measured at 570 nm.

### Confocal microscopy

Huh7 cells (4 × 10^4^) were seeded onto coverslips and treated as indicated. The cells were fixed in 4% PFA at room temperature for 30 min and blocked with 0.1% Triton X-100 (diluted in PBS) and 1% BSA for 10 min. The cells were then incubated with anti-HMGB1 antibody (1:300, ab18256, Abcam) overnight at 4°C and with rhodamine-labeled anti-rabbit IgG (1:300, 143,572, Jackson ImmunoRes Lab) at room temperature for 1 h. Subsequently, the cells were stained with DAPI (1:10,000; 0100–20, Southern Biotech) at room temperature for 5 min and covered with glass slides. All slides were examined under the Zeiss LSM 700 laser scanning confocal microscope and the results were assessed using the ZEN software.

### Enzyme-linked immunosorbent assay

Liver tissues were mixed with 0.9% saline, homogenized and then centrifuged to obtain the supernatant. The levels of TNF-α, IL-1β, IL-6, and COX-2 were determined by enzyme-linked immunosorbent assay (ELISA) according to the manufacturer’s instructions (Shanghai Jianglai Biological Technology, Shanghai, China). The absorbance was measured at 450 nm using a microplate reader.

### Western blotting

Western blotting was performed using total protein samples extracted from mouse liver and serum as well as cell lines. The amount of protein in the samples was quantified using Pierce™ BCA protein assay kit (23,227, Thermo Fisher Scientific). Subsequently, the total proteins were resolved by SDS-PAGE and transferred onto polyvinylidene difluoride membranes. After blocking the membranes at room temperature for 1 h, they were incubated at 4°C overnight with the following primary antibodies: anti-β-actin (1:1000, 4,970, Cell Signaling Technology), anti-HMGB1 (1:1000, ab18256, Abcam), anti-albumin (1:1000, ab207327, Abcam), anti-NF-κB (1:1000, ab16502, Abcam), anti-TLR-4 (1:500, ab13556, Abcam), anti-MyD88 (1:1000, ab219413, Abcam) and anti-lamin B (1:1000, sc-6216, Santa Cruz Biotechnology). The membranes were then incubated with anti-rabbit IgG antibody (1:2000, ab6721, Abcam) for 1 h at room temperature. The protein bands were visualized using the ECL detection reagent (32,106, Thermo Fisher Scientific) and quantified using the Quantity One Software (Bio-Rad).

### Statistical analysis

Results were expressed as mean ± standard deviation. GraphPad Prism eight was used for statistical analyses. All *p-*values were calculated by one-way analysis of variance, followed by Tukey’s post hoc test; *p-*values of <0.05 indicated statistical significance.

## Results

### Levels of high mobility group box 1 in the liver and serum of the LPS/D-GalN-induced ALF mouse model

**FIGURE 1:**
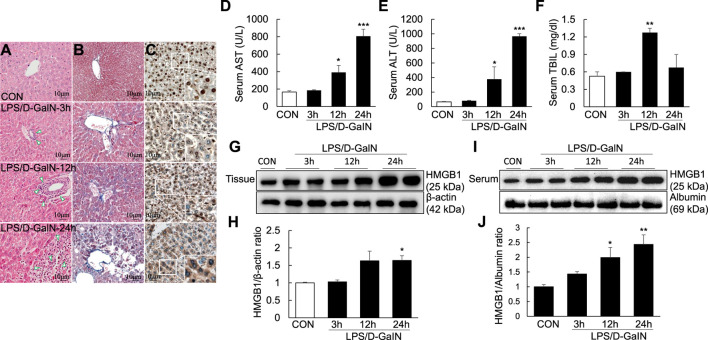
ALI and extracellular levels of HMGB1 in the LPS/D-GaIN-induced ALF mouse model. LPS (10 μg/kg) and D-GalN (600 mg/kg) were administered intraperitoneally (i.p.) to BALB/c mice for 3, 12, and 24 h. We determined the extent of inflammatory cell infiltration **(A)** as well as liver cell necrosis and fibrosis **(B)** *via* H and E and Masson staining. Extracellular levels of HMGB1 were determined *via* immunohistochemistry **(C)**. Representative images and results are presented as means ± SEM (*n* = 3). We measured AST, ALT, and TBIL levels in the serum **(D–F)** and examined HMGB1 levels in the liver **(G,H)** and serum **(I,J)** by western blotting (*n* = 3). *p < 0.05, **p < 0.01, ***p < 0.001 vs. control.

Pathological changes in the liver; AST, ALT, and TBIL levels; and other biochemical indicators are important markers for evaluating liver injury. H and E and Masson staining of the liver sections were performed to observe necrosis, inflammatory cell infiltration and liver fibrosis progression in the liver tissue. H and E staining revealed the presence of a large number of inflammatory cells in the portal area of the liver and around the central vein (as shown by the arrow) at 3, 12, and 24 h after LPS/D-GaIN administration; the degree of inflammatory cell infiltration increased with time (Figure 1A). Masson staining revealed that fibrosis was significantly increased in the liver tissues at 12 h after LPS/D-GaIN administration and severe hepatocyte necrosis was observed at 24 h after LPS/D-GaIN administration (Figure 1B). Furthermore, the serum AST and ALT levels in ALF mice were significantly increased at 12 and 24 h after LPS/D-GaIN administration (Figures 1D,E). TBIL levels also increased with time, reaching a peak at 12 h (Figure 1F). These results indicated that LPS/D-GaIN could induce ALF in mice.

Activated HMGB1 has been reported to play a key role in the pathogenesis of ALF (Yamamoto and Tajima, 2017). However, it remains to be verified whether it acts after it is released extracellularly. Immunohistochemistry using anti-HMGB1 antibody revealed excessive HMGB1 in the nuclei of mouse hepatocytes. At 3 and 12 h after LPS/D-GaIN administration, HMGB1 activity was detected in both the nuclei and cytoplasm of mouse hepatocytes. However, at 24 h after LPS/D-GaIN administration, HMGB1 activity was mainly detected in the cytoplasm, with only a small amount of HMGB1 being expressed in the nucleus (Figure 1C). Immunoblotting revealed that compared with the control, the expression of HMGB1 was significantly upregulated in the mouse liver tissues (Figures 1G,H) and serum (Figures 1I,J) at 24 h after LPS/D-GaIN administration. LPS/D-GaIN administration could induce the extracellular release of HMGB1.

### Upregulation of TLR-4/NF-κB signaling and proinflammatory cytokines in the LPS/D-GalN-induced ALF mouse model

**FIGURE 2:**
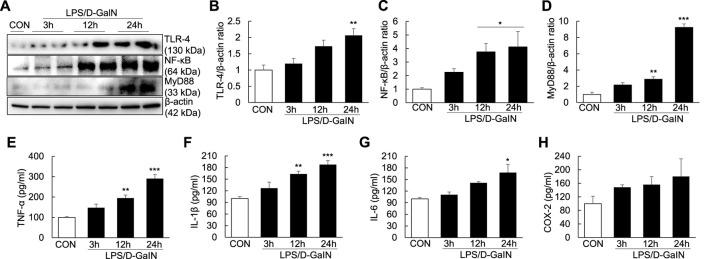
Expression of TLR-4/NF-κB signaling molecules and proinflammatory cytokines in the LPS/D-GaIN-induced ALF mouse model. LPS (10 μg/kg) and D-GalN (600 mg/kg) were administered intraperitoneally (i.p.) to BALB/c mice for 3, 12, and 24 h. We examined TLR-4, NF-κB and MyD88 levels in the liver using western blotting **(A–D)**. The levels of proinflammatory cytokines (TNF-α, IL-1β, and IL-6) and COX-2 in the serum were determined using ELISA **(E–H)** (*n* = 3). *p < 0.05, **p < 0.01, ***p < 0.001 vs. control.

To explore whether inflammatory injury is an important factor for inducing and aggravating ALF, we measured the levels of TLR-4/NF-κB signaling molecules and proinflammatory cytokines in the LPS/D-GalN-induced ALF mouse model. Compared with treatment-naïve controls, the expression of TLR-4, NF-κB, and MyD88 in the liver tissues of treated mice was significantly upregulated at 12 and 24 h after LPS/D-GaIN administration (Figures 2A–D). Moreover, the levels of TNF-α, IL-1β, and IL-6 in the serum were significantly increased in ALF mice than in treatment-naïve controls at 12 and 24 h after LPS/D-GaIN administration (Figures 2E–G). The levels of COX-2 in the serum were increased in ALF mice than in treatment-naïve controls, albeit not significantly (Figure 2H). These results indicated that systemic inflammatory injury in the ALF model exacerbated in both liver and serum at 12 and 24 h after LPS/D-GalN administration in the ALF mouse model and that this effect was dependent on TLR-4/NF-κB signaling.

### high mobility group box 1-A box suppresses the extracellular release of high mobility group box 1 and TLR-4/NF-κB signaling in the LPS/D-GalN-induced ALF mouse model

**FIGURE 3:**
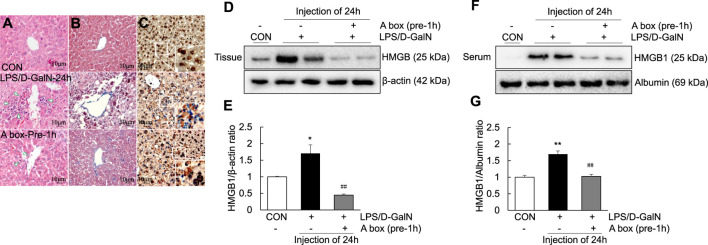
Decrease in ALI and extracellular levels of HMGB1 in the LPS/GaIN-induced ALF mouse model following HMGB1-A box administration. BALB/c mice were administered HMGB1-A box (400 μg, i.p.) 1 h before LPS (10 μg/kg) and D-GalN (600 mg/kg) administration and were sacrificed 24 h after the ALF mouse model was established. We used H&E staining to determine the extent of inflammatory cell infiltration **(A)**, Masson staining to determine the extent of liver cell necrosis and fibrosis **(B)** and immunohistochemistry to detect the extracellular release of HMGB1 **(C)**. Representative images and results are presented as means ± SEM (*n* = 3). We examined HMGB1 levels in the liver **(D,E)** and serum **(F,G)** by western blotting (*n* = 3). *p < 0.05, **p < 0.01 vs. control, ^##^ *p* < 0.01 vs. LPS group.

**FIGURE 4:**
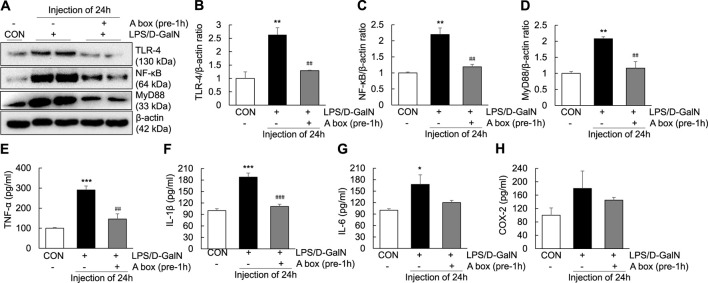
Inhibition of TLR-4/NF-κB signaling and proinflammatory cytokine expression in the LPS/GaIN-induced ALF mouse model following HMGB1-A box administration. BALB/c mice were administered HMGB1-A box (400 μg, i.p.) 1 h before LPS (10 μg/kg) and D-GalN (600 mg/kg) administration and were sacrificed 24 h after the ALF mouse model was established. We examined the levels of TLR-4, NF-κB and MyD88 in the liver by western blotting **(A–D)** and the levels of proinflammatory cytokines (TNF-α, IL-1β and IL-6) and COX-2 in the serum by ELISA **(E–H)** (*n* = 3). *p < 0.05, **p < 0.01, ***p < 0.001 vs. control, ^##^ *p* < 0.01, ^###^ *p* < 0.001 vs. LPS group.

To explore whether HMGB1-A box can reduce liver inflammation by inhibiting the extracellular release of HMGB1, HMGB1-A box was administered at 1 h before LPS/D-GalN administration (24 h, i.p.) and liver injury was evaluated at the same time as in the LPS/D-GalN-induced ALF mouse model (Figures 3A,B). H and E and Masson staining of the liver sections were performed to observe inflammatory cell infiltration (as shown by the arrow in Figure 3A) as well as liver cell necrosis and fibrosis (Figure 3B), which were found to be significantly reduced after treatment with HMGB1-A box (400 μg, i.p.). Moreover, the administration of HMGB1-A box (400 μg, i.p.) in ALF mice inhibited the transfer of HMGB1 from the nucleus to the serum in the LPS/D-GalN-induced ALF mouse model (Figures 3C–G). TLR-4, NF-κB, and MyD88 levels in the liver (Figures 4A–D) and TNF-α and IL-1β levels in the serum (Figures 4E,F) were significantly decreased after HMGB1-A box (400 μg, i.p.) administration in ALF mice compared with those in the LPS/D-GalN-induced ALF mouse model. The levels of IL-6 and COX-2 in the serum tended to decrease, albeit not significantly, after HMGB1-A box administration in ALF mice compared with those in the LPS/D-GalN-induced ALF mouse model (Figures 4G,H). The levels of IL-6 and COX-2 in the serum showed the same trend as the levels of TNF-α and IL-1β. These results suggested that HMGB1-A box can interfere with a series of inflammatory responses involved in ALF by inhibiting the extracellular release of HMGB1.

### Induction of cell apoptosis and release of proinflammatory cytokines in LPS-treated Huh7 cells

**FIGURE 5:**
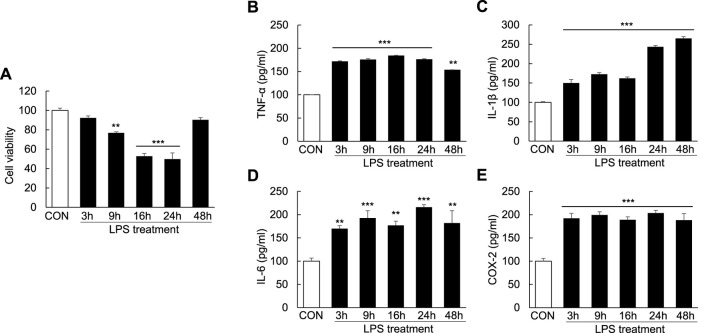
Induction of cell apoptosis and proinflammatory cytokines in LPS-induced Huh7 cells. Huh7 cells were treated with LPS (1000 ng/ml) for 3, 9, 16, 24, and 48 h. MTT assay **(A)** and ELISA **(B–E)** were conducted 24 h after LPS treatment. **p < 0.01, ***p < 0.001 vs. control.

To examine cell apoptosis and inflammation following LPS induction, Huh7 cells were treated with LPS (1000 ng/ml) for 3, 9, 16, 24, and 48 h. Compared with controls, cell viability was reduced to 23.2% ± 1.15% in the LPS-treated cells at 9 h, indicating the initiation of apoptosis; the degree of cell apoptosis remained significant at 16 and 24 h after LPS treatment (Figure 5A). Moreover, the levels of the proinflammatory cytokines TNF-α, IL-1β, and IL-6 as well as COX-2 were markedly increased after LPS treatment for 3, 9, 16, 24, and 48 h (Figures 5B–E). These results showed that LPS could induce cell apoptosis and proinflammatory cytokine release in Huh7 cells.

### Upregulation of the extracellular release of high mobility group box 1 and TLR-4/NF-κB signaling in LPS-induced Huh7 cells

**FIGURE 6:**
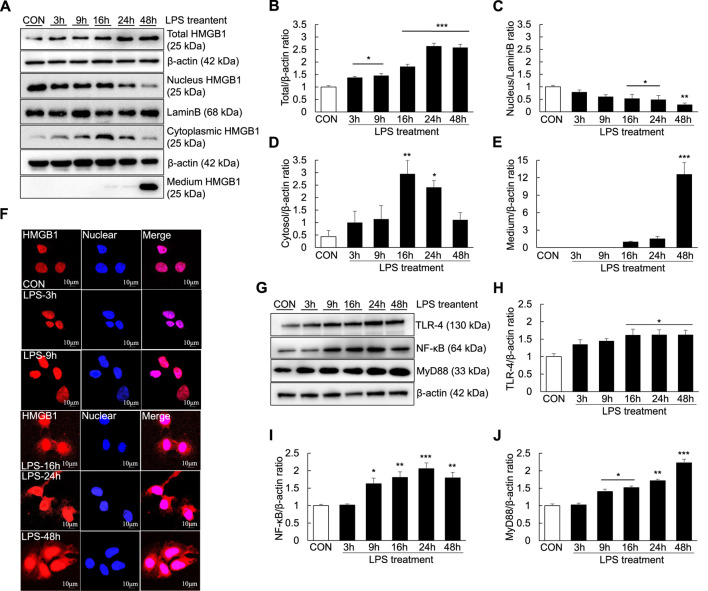
Extracellular release of HMGB1 and TLR-4/NF-κB signaling in LPS-induced Huh7 cells. Huh7 cells were treated with 1000 ng/ml LPS for 3, 9, 16, 24, and 48 h. Western blotting was performed to determine the total, nuclear and cytoplasmic levels of HMGB1 **(A–D)** as well as the extracellular levels of HMGB1 (in the medium) **(A,E)**. Confocal microscopy was performed using anti-HMGB1 antibody and DAPI **(F)** and HMGB1-positive cells were identified using an anti-rhodamine-conjugated antibody. We also examined TLR-4,NF-κB, and MyD88 expression *via* western blotting **(G–J)**. *p < 0.05, **p < 0.01, ***p < 0.001 vs. control.

As extracellular HMGB1 can aggravate the inflammatory response by upregulating TLR-4/NF-κB signaling and proinflammatory cytokine release in the ALF mouse model, we investigated whether LPS could induce inflammation and the extracellular release of HMGB1 in Huh7 cells. The temporal profiles of LPS-induced upregulation of HMGB1 in Huh7 cells were examined by western blotting and confocal microscopy after LPS treatment. LPS treatment (1000 ng/ml) for 3 h significantly increased the total HMGB1 levels, which further increased until 48 h (Figures 6A,B). Cytoplasmic HMGB1 was detected after LPS treatment for 3 h, the levels of which further increased until 16 and 24 h (Figures 6A–D). Conversely, compared with controls, the levels of nuclear HMGB1 decreased in the cells treated with LPS for 16–48 h (Figures 6A–C).

Furthermore, after 16 h of LPS treatment, a small amount of HMGB1 was present in the culture medium. HMGB1 levels in the culture medium substantially increased after 48 h of LPS treatment, indicating that HMGB1 was completely transferred from the nucleus and released extracellularly (Figures 6A–E). However, the results of confocal microscopy did not entirely corroborate those of western blotting. Cytoplasmic HMGB1 was detected after 16 h of LPS treatment and its levels peaked after 48 h (Figure 6F). Moreover, TLR-4, NF-κB and MyD88 levels were increased after LPS treatment: TLR-4 levels started to increase after 16 h of LPS treatment, whereas NF-κB levels started to increase after 9 h of LPS treatment (Figures 6G–J).

### high mobility group box 1-A box inhibits cell apoptosis and proinflammatory cytokines in LPS-induced Huh7 cells

**FIGURE 7:**
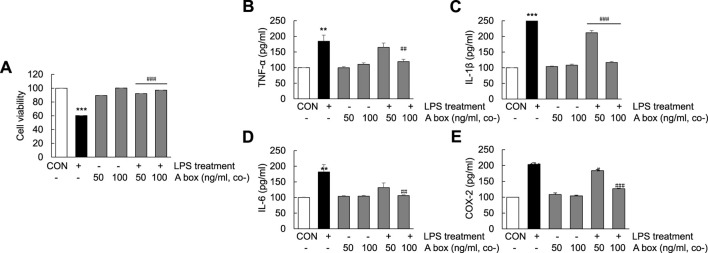
Suppression of cell apoptosis and decrease in proinflammatory cytokine levels in LPS-induced Huh7 cells following HMGB1-A box treatment. Huh7 cells were treated HMGB1-A box (50 and 100 ng/ml) only for 24 h without LPS treatment and LPS (1000 ng/ml) for 24 h with or without HMGB1-A box (50 and 100 ng/ml). MTT assay was performed to measure cell viability **(A)**. ELISA was performed to assess the expression of proinflammatory cytokines (TNF-α, IL-1β, and IL-6) and COX-2 (**B–E)**. ***p < 0.001 vs. control, ^#^ *p* < 0.05, ^##^ *p* < 0.01, ^###^ *p* < 0.001 vs. LPS group.

The protective effect of HMGB1-A box in the liver against ALF-induced liver injury prompted us to examine whether HMGB1-A box exerts the same effect in Huh7 cells. The viabilities of Huh7 cells treated with HMGB1-A box (50 or 100 ng/ml) without LPS for 24 h were no difference compared with controls and cotreated with HMGB1-A box (50 or 100 ng/ml) and LPS (1000 ng/ml) for 24 h were significantly increased (21.4% ± 0.01% and 29.3% ± 0.00%, respectively) compared with those of Huh7 cells treated with LPS alone (Figure 7A). Furthermore, the levels of TNF-α, IL-1β, IL-6, and COX-2 also were no changes with HMGB1-A box treatment only and effectively decreased after HMGB1-A box treatment compared with LPS treatment in Huh7 cells (Figures 7B–E). These results indicated that HMGB1-A box plays a protective role against LPS-induced cell apoptosis and inflammation in Huh7 cells.

### high mobility group box 1-A box inhibits the extracellular release of high mobility group box 1 and TLR-4/NF-κB signaling in LPS-induced Huh7 cells

**FIGURE 8:**
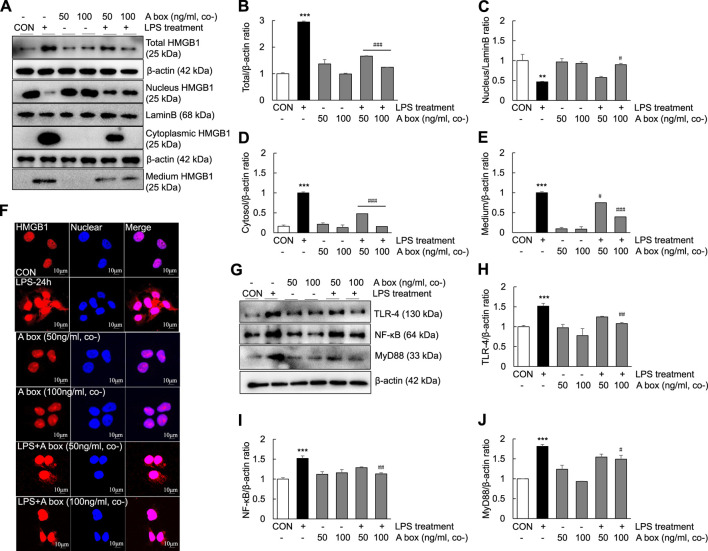
Suppression of the extracellular release of HMGB1 and TLR-4/NF-κB signaling in LPS-induced Huh7 cells following HMGB1-A box treatment. Huh7 cells were treated HMGB1-A box (50 and 100 ng/ml) only for 24 h without LPS treatment and LPS (1000 ng/ml) for 24 h with or without HMGB1-A box (50 and 100 ng/ml). Western blotting was performed to determine the total, nuclear, cytoplasmic and extracellular levels of HMGB1 **(A–E)**. Confocal microscopy was performed using anti-HMGB1 antibody and DAPI to determine the extracellular release of HMGB1 **(F)**. Western blotting was performed to assess the levels of TLR-4, NF-κB and MyD88 **(G–J)**. *p < 0.05, **p < 0.01 vs. control, ^#^ *p* < 0.05, ^##^ *p* < 0.01, ^###^ *p* < 0.001 vs. LPS group.

To determine whether HMGB1-A box plays a role in reducing inflammation by inhibiting the extracellular release of HMGB1, we investigated the levels of extracellular HMGB1 and the expression of TLR-4/NF-κB signaling molecules in LPS-induced Huh7 cells (Figure 8). The levels of total HMGB1 were significantly decreased in the cells cotreated with 50 ng/ml HMGB1-A box and 1000 ng/ml LPS for 24 h than in those treated with 1000 ng/ml LPS alone; these levels decreased further in the cells cotreated with 100 ng/ml HMGB1-A box (Figures 8A,B). The levels of cytoplasmic and extracellular HMGB1 were also markedly decreased after cotreatment with 50 ng/ml HMGB1-A box, which decreased further after cotreatment with 100 ng/ml HMGB1-A box (Figures 8A,D,E). Conversely, the levels of nuclear HMGB1 were significantly increased in the cells cotreated with 100 ng/ml HMGB1-A box and 1000 ng/ml LPS for 24 h compared with those treated with LPS alone (Figures 8A–C). Confocal microscopy revealed that the extracellular release of HMGB1 was markedly inhibited in the cells treated with 50 or 100 ng/ml HMGB1-A box for 24 h compared with those treated with 1000 ng/ml LPS for 24 h (Figure 8F). Similarly, the levels of TLR-4, NF-κB and MyD88 decreased after cotreatment with HMGB1-A box (50 or 100 ng/ml) for 24 h (Figures 8G–J). These results indicated that cotreatment with HMGB1-A box could effectively inhibit the extracellular release of HMGB1 as well as TLR-4/NF-κB signaling in LPS-induced Huh7 cells.

## Discussion

ALF is a clinical syndrome primarily characterized by large-scale hepatocyte necrosis and inflammatory cell infiltration of varying degrees, which is caused by a variety of factors, such as hepatitis viruses, drugs and hepatotoxic substances (Lefkowitch, 2016; Flamm et al., 2017; Wendon et al., 2017). Owing to its rapid onset, multiple complications and high mortality, it has become a serious threat to human health (Bernal and Wendon, 2013; Lefkowitch, 2016; Flamm et al., 2017). Liver transplantation is currently considered an effective treatment, but its clinical application is limited by various factors, such as the lack of liver donors, postoperative complications and immune rejection (O'Grady, 2014; Germani et al., 2012). Moreover, the lack of specific drugs is the primary reason for the high mortality associated with ALF (Lefkowitch, 2016). Several studies have shown that inflammation is a key factor in the processes involved in ALF progression, such as the activation of inflammatory signaling (Xia et al., 2015; Zhang et al., 2020) and the upregulation of proinflammatory cytokines such as TNF-α, IFN-9, and IL-1 (Streetz et al., 2000; Wu et al., 2010). It has also been reported that the inflammatory mediator HMGB1 can induce the development and progression of ALF (Majumdar et al., 2013; Basta et al., 2015; He et al., 2015). In our study, we observed massive necrosis (Figure 1B) and inflammatory cell infiltration (Figure 1A) in the liver of the LPS/D-GaIN-induced ALF mouse model. The expression of HMGB1 in the liver and serum was also significantly increased (Figures 1G–J). Therefore, we speculated that HMGB1 induces a series of inflammatory processes in the LPS/D-GaIN-induced ALF mouse model.

As a nonhistone nucleoprotein, HMGB1 is widely present in the cells of various mammalian organs, such as the brain (Dehghani et al., 2021), lungs (Chen et al., 2021), heart (Pellegrini et al., 2019), liver (Arshad et al., 2012; Zhang et al., 2020), kidneys (Chen et al., 2017) and spleen (Valdes-Ferrer et al., 2013). Under normal physiological conditions, HMGB1 is localized in the nucleus of most cells and its structure is highly conserved. It can bind to DNA and plays an essential role in promoting DNA folding, translation and replication as well as stabilizing the chromosome structure (Mandke and Vasquez, 2019; Wang et al., 2021). When the body is stimulated or damaged, HMGB1 is released extracellularly as a stress signal and inflammatory mediator, where it participates in a series of inflammatory responses in the body as an effective inflammatory signaling molecule (Zhou et al., 2018; Deng et al., 2021; Liu et al., 2021). Studies have demonstrated that when HMGB1 is stimulated by inflammatory mediators or endotoxins, the lysine in the nuclear localization signal sequence of HMGB1 is acetylated, resulting in its translocation from the nucleus to the extracellular matrix (Rabadi et al., 2012; Wang et al., 2019). Extracellular HMGB1 can bind to receptors on different surfaces and accelerate the process of inflammation by activating relevant molecules in downstream signaling pathways, such as target receptors TLR-2/4 and receptor for advanced glycation endproducts (RAGE) (Wang et al., 2019; Li et al., 2020). HMGB1 has also been reported to induce and promote inflammatory responses through interaction with a variety of molecules, including LPS. The binding of LPS to HMGB1 could induce NO production in macrophages (Chakraborty et al., 2013) and activate NF-κB and MAPK signaling *via* binding to TLR-4 and RAGE (Youn et al., 2008; Chakraborty et al., 2013). In this study, the levels of TLR-4, NF-κB and MyD88 in the liver (Figures 2A–D) as well as the levels of COX-2 and proinflammatory cytokines (TNF-α, IL-1β, and IL-6) in the serum (Figures 2D–G) increased over time after LPS/D-GaIN administration in the ALF mouse model. These results were consistent with the expression of HMGB1 in the liver and serum (Figures 1G–J). Therefore, it can be assumed that extracellular HMGB1 aggravates liver injury by activating TLR-4/NF-κB signaling and increasing the expression of proinflammatory cytokines in the LPS/D-GaIN-induced ALF mouse model. We also demonstrated that LPS could induce cell apoptosis (Figure 5A) and proinflammatory cytokine expression (Figures 5B–E) in Huh7 cells. The levels of HMGB1, TLR-4, NF-κB, and MyD88 also increased in a time-dependent manner following LPS treatment (Figures 6A,B,G–J). In particular, HMGB1 started to gradually translocate from the nucleus to the cytoplasm at 3 h after LPS treatment. Subsequently, the levels of HMGB1 in the cytoplasm peaked at 16 h after LPS treatment. Further, at this time point, HMGB1 could be detected in the culture medium. The levels of HMGB1 in the culture medium peaked at 48 h after LPS treatment, indicating that HMGB1 had been completely translocated from the nucleus to the outside of the cell (Figures 6A,B). However, the results of confocal microscopy were slightly different, in which this translocation of HMGB1 could not be detected until 16 h after LPS treatment (Figure 6F). This is consistent with our results, which may be attributed to the time-dependent increase in the expression of HMGB1 and the number of cells releasing HMGB1 extracellularly. The *in vitro* results also suggested that LPS-induced cell apoptosis was related to the inflammatory response and the extracellular release of HMGB1.

HMGB1 contains 215 amino acid residues. Starting from the amino-terminus to the carboxyl-terminus, 1–79 amino acid residues represent the A box, 89–162 amino acid residues represent the B box and 186–215 amino acid residues, which are mainly aspartic acid and glutamic acid residues, represent the acidic C-terminus (Harris et al., 2012). Examination of the structure and function of HMGB1 revealed that the B box is a functional proinflammatory domain. The A box is similar to the B box in structure, but it is not proinflammatory. Instead, it antagonizes inflammation. In other words, HMGB1-A box antagonizes HMGB1-B box (Hwang et al., 2017). In several inflammation-related studies, recombinant HMGB1-A box has been successfully used as an antagonist of HMGB1 to alleviate and treat related inflammatory damage (Yuan et al., 2009; Kikuchi et al., 2014; Han et al., 2015; Wang et al., 2015). In the present study, HMGB1-A box was used as an effective anti-inflammatory agent to inhibit a series of inflammatory reactions involved in ALF, thereby reducing the extensive necrosis of hepatocytes and the infiltration of inflammatory cells in the ALF mouse liver (Figure 3) and inhibiting the expression of TLR-4/NF-κB signaling molecules and related proinflammatory cytokines (Figure 4). HMGB1-A box also played an important role in inhibiting cell apoptosis, inflammatory signaling and proinflammatory cytokine expression in LPS-induced Huh7 cells (Figures 7, 8). However, it was not previously clarified whether it acts by inhibiting the activation of HMGB1 and/or preventing the extracellular release of HMGB1. We found that after the administration of HMGB1-A box, extranuclear HMGB1 levels significantly reduced (Figure 3C). Similarly, serum HMGB1 levels were significantly lower in rats treated with HMGB1-A box than in the ALF model controls (Figures 3F,G). In addition, HMGB1 levels were particularly low in the cytoplasm and culture media after the cotreatment of Huh7 cells with HMGB1-A box and LPS (Figures 8A,D,E). Confocal microscopy results further confirmed that HMGB1-A box could effectively prevent the translocation of HMGB1 outside the nucleus (Figure 8F).

## Conclusion

HMGB1-A box, a specific antagonist of HMGB1, played a protective role by inhibiting inflammatory liver injury through the regulation of HMGB1/TLR-4/NF-κB signaling in the LPS/D-GaIN-induced ALF mouse model. Altogether, this study demonstrated that HMGB1-A box inhibited the development of ALF, which may be related to preventing the extracellular release of HMGB1.

## Data Availability

The original contributions presented in the study are included in the article/supplementary materials, further inquiries can be directed to the corresponding authors.

## Abbreviations

ALF, acute liver failure; ALI, acute liver injury; HMGB1, high mobility group box one; ALT, alanine aminotransferase; AST, aspartate aminotransferase; DMEM, Dulbecco’s modified Eagle medium; ELISA, enzyme-linked immunosorbent assay; H and E, hematoxylin and eosin; TBIL, total bilirubin.
